# Sustaining Gains Made in Voluntary Medical Male Circumcision

**DOI:** 10.9745/GHSP-D-16-00106

**Published:** 2016-07-02

**Authors:** Chewe Luo

**Affiliations:** a United Nations Children’s Fund (UNICEF), New York, NY, USA

## Abstract

Introducing early infant male circumcision (EIMC) can sustain voluntary medical male circumcision (VMMC) programs. This *Global Health: Science and Practice* supplement presents lessons learned, research findings on demand creation, and cost comparisons of various models of EIMC introduction.

Fourteen countries in sub-Saharan Africa with high HIV burden and low prevalence of male circumcision are currently providing voluntary medical male circumcision (VMMC) services in line with the 2007 recommendations from the World Health Organization (WHO) and the Joint United Nations Programme on HIV/AIDS (UNAIDS). VMMC, which reduces the risk of heterosexually acquired HIV infection in men by approximately 60%,^1^^-^^3^ is one element of a combination HIV-prevention package that can include correct and consistent use of condoms, HIV testing and counseling, treatment of sexually transmitted infections, behavior change communication, harm reduction for people who inject drugs, community-based interventions for key populations, antiretroviral treatment for people living with HIV, and pre- and post-exposure prophylaxis.

The main thrust of VMMC programs has been to circumcise men ages 15 to 49 years to reduce their risk of HIV infection. Scaling up VMMC to reach the target of 80% coverage among men ages 15 to 49 years in the 13 countries would mean that approximately 20 million circumcisions would need to have been performed by the end of 2015. This would have averted 3.4 million new HIV infections over 15 years, 22% of the total projected infections.^4^ As of December 2015, more than 10 million circumcisions had been performed, and some regions had already reached the saturation threshold of 80% coverage.^5^ Settings that reached 80% coverage are starting to plan how to sustain this coverage and maintain the population-level prevention impact and individual-level gains.

## A TWO-PHASE APPROACH TO VMMC SCALE-UP

The 2007 WHO–UNAIDS Joint Strategic Action Framework lays the foundation of a two-phase approach to scaling up VMMC: the catch-up phase that focuses on reaching at least 80% of VMMC prevalence among 15- to 49-year-old men and the sustainability phase.^6^ Three sustainability scenarios have been identified in the framework, depending on whether a country introduces and scales up early infant male circumcision (EIMC). EIMC is medical male circumcision performed between 12–24 hours and 60 days after birth.^7^

In the first sustainability scenario, the country maintains high circumcision coverage among the adolescent population (ages 10 to 19 years) and does not introduce EIMC. The second scenario is to scale up EIMC while maintaining high coverage among adolescents, and when 80% of the cohort of circumcised infants reaches 10 to 14 years of age, the focus shifts entirely to EIMC. The third scenario is to introduce EIMC but continue circumcision of both adolescents and infants indefinitely. EIMC has several advantages over adolescent and adult VMMC: it costs less, the surgery heals more quickly and has less risk of adverse events, and it can be integrated into routine maternal, newborn, and child health platforms.

Three sustainability scenarios have been identified depending on whether a country introduces and scales up early infant male circumcision.

## INTRODUCING EIMC

Integrating a one-time surgical intervention with long-term benefits into routine health care packages is not straightforward. Although interventions for both the mother and infant delivered at the same time in the same place by the same health care provider or team save more lives and improve health outcomes of mothers and infants better than providing services separately, there are many policy, programmatic, and logistical issues to consider.^8^ The VMMC community needs to act cautiously to avoid negative events and avoid contributing to newborn mortality. Mortality in newborns from all causes currently accounts for 44% of all deaths among children less than 5 years of age.

Integrating a one-time surgical intervention with long-term benefits such as EIMC into routine health care packages is not straightforward.

The *Every Newborn* action plan was developed in response to country demand, and based on the latest epidemiology and evidence, it outlines specific actions to improve access to and improve the quality of health care for women and newborns within the continuum of care.^8^ Although there is no ideal time identified for performing medical male circumcision during infancy, the American Academy of Family Physicians recommends waiting until at least 12–24 hours after birth to ensure the infant is stable.^9^ This critical time enables providers to assess the newborn and check for contraindications to circumcision. On the other hand, no added risks of EIMC have been reported. National governments considering introducing medical male circumcision for young infants should look at local data and decide on the optimal time for the intervention.

This supplement to *Global Health: Science and Practice*, sponsored by the United Nations Children’s Fund (UNICEF) and the U.S. President’s Emergency Plan for AIDS Relief (PEPFAR), brings together several articles on EIMC, including reviews that highlight lessons learned and challenges of providing circumcision services to young male infants; formative research findings on the perceptions of parents, providers, and other stakeholders to inform demand creation for EIMC; and cost comparisons of alternative service delivery models as a result of task shifting and integration into newborn care. The supplement also includes a commentary on an important consideration for successful and sustainable national EIMC programs: service delivery through maternal, newborn, and child health service platforms.

Prevention is a cornerstone of the momentum toward ending AIDS by 2030, following the UNAIDS *Fast-Track* strategy. VMMC is a proven, effective prevention approach and a component of combination prevention packages. This supplement provides country experiences on scaling up as well as options for sustaining efforts in VMMC and their cost benefits.
